# Predictive Factors for Prolonged Ventilatory Support in Infants Undergoing Total Anomalous Pulmonary Venous Connection Repair: A Retrospective Cohort Study

**DOI:** 10.31083/RCM39215

**Published:** 2025-10-20

**Authors:** Chun-xiang Li, Xiao-lei Gong, Li-min Zhu, Xin-wei Du, Hai-bo Zhang, Zhuo-ming Xu

**Affiliations:** ^1^Department of Cardiac Intensive Care Unit, Heart Center, Shanghai Children’s Medical Center, Shanghai Jiaotong University School of Medicine, 200127 Shanghai, China; ^2^Cardiovascular and Thoracic Surgery Department, Heart Center, Shanghai Children’s Medical Center, Shanghai Jiaotong University School of Medicine, 200127 Shanghai, China

**Keywords:** total anomalous pulmonary venous connection, prolonged recovery, pulmonary venous flow, left ventricular end diastolic dimension

## Abstract

**Background:**

Total anomalous pulmonary venous connection (TAPVC) is a congenital heart defect requiring surgical correction and is associated with significant postoperative risks, such as prolonged ventilatory support and mortality. This study aimed to identify perioperative factors that contribute to protracted ventilatory support in infants undergoing TAPVC repair.

**Methods:**

Infants aged under 6 months with TAPVC who underwent primary surgical repair between January 2017 and December 2022 were retrospectively analyzed. Patients were divided into two groups based on the duration of postoperative ventilatory support: group A (prolonged recovery, with ventilatory support durations exceeding the 75th percentile) and group B (normal recovery). Perioperative characteristics between the groups were compared using various statistical methods, including multivariate logistic regression.

**Results:**

A total of 323 children were analyzed, with 66 and 257 children in groups A and B, respectively. The median duration of ventilatory support and intensive care unit (ICU) stay was significantly longer in group A (182 hours and 12 days) compared to group B (52 hours and 5.5 days). Multivariate logistic regression analysis identified the following as independent risk factors for prolonged recovery period: weight-for-age Z score <–2 (*p* = 0.022), preoperative pulmonary venous obstruction (*p* = 0.042), emergency surgery (*p* = 0.043), prolonged cardiopulmonary bypass duration and aortic cross-clamp (ACC) time (*p* = 0.014), diaphragmatic injury for plication (*p* = 0.045), and velocity of pulmonary venous flow/left ventricular end diastolic dimension (PV/LVDD) ratio (*p* = 0.012). These factors individually increase the likelihood of delayed recovery by 6.4-fold, 6-fold, 5.9-fold, 8.6-fold, 5.3-fold, and 9.8-fold, respectively.

**Conclusion:**

While most infants recover suitably after TAPVC repair, those with a weight-for-age Z score <–2, preoperative pulmonary venous obstruction, emergency surgery, prolonged cardiopulmonary bypass and aortic cross-clamp time, diaphragmatic injury, and a PV/LVDD ratio >0.624 are at a higher risk for prolonged recovery. Early identification of these factors can help optimize perioperative management and improve outcomes.

## 1. Introduction

Total anomalous pulmonary venous connection (TAPVC) is a rare but heterogeneous anomaly, which accounts for 1%–3% of all congenital heart disease cases [1, 2, 3]. Pulmonary venous obstruction (PVO) has been reported in 0%–18% of patients undergoing TAPVC repair [4]. The perioperative mortality of TAPVC has decreased significantly with advancements in diagnostic accuracy, surgical techniques, and perioperative management. Nevertheless, the early mortality rate over the past decade has remained as high as 10.7% [5, 6]. Early postoperative deaths are related to the age of children, postoperative PVO, preoperative circulatory instability, and mixed anatomical variations [7]. Children with TAPVC are often complicated by PVO, severe pulmonary hypertension (PH), and are prone to metabolic acidosis, which requires emergent surgery and carries a high risk of delayed recovery or even death. Thus, this study conducted a retrospective analysis of a large cohort of children with TAPVC to identify perioperative factors associated with delayed recovery and to optimize management strategies for high-risk patients.

## 2. Methods

### 2.1 Study Design and Participant Selection

We conducted a retrospective cohort study in the Cardiac Intensive Care Unit (CICU) of the Heart Center at Shanghai Children’s Medical Center between January 2017 and December 2022. The hospital Institutional Review Board approved the study protocol.

**Fig. 1.**
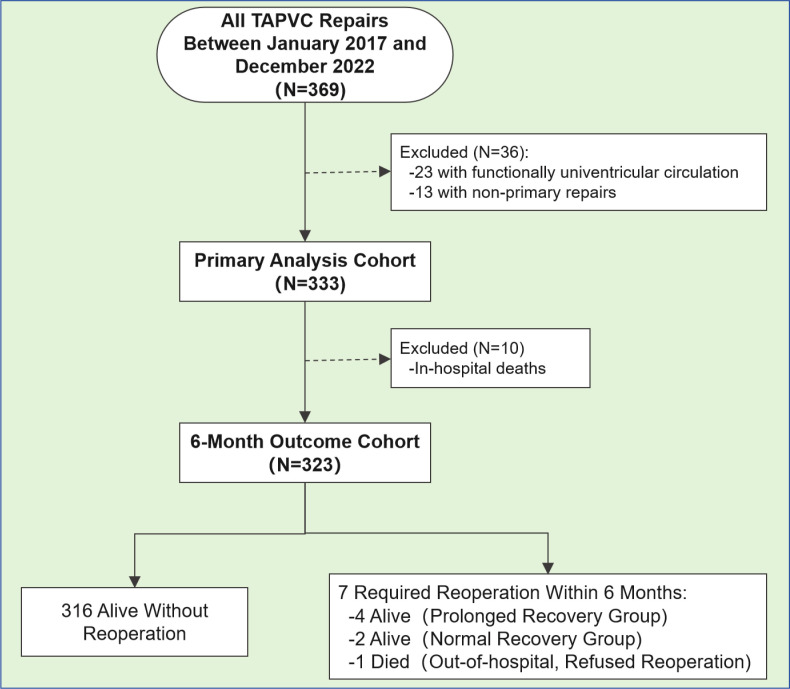
**Patient flowchart**. TAPVC, total anomalous pulmonary venous connection. A total of 369 children received a TAPVC repair during the study period. A total of 323 children were included in the analysis.

This study included children aged under 6 months who underwent primary surgical repair for TAPVC. Exclusion criteria included secondary repair, functionally univentricular circulation, in-hospital death, or incomplete medical records. Participants were stratified into two groups based on the duration of postoperative ventilatory support. Children with ventilatory support durations exceeding the 75th percentile were classified into the delayed recovery group (group A). In contrast, the remaining children were assigned to the normal recovery group (group B) (Fig. 1). No changes were made to the devices used, the medical teams, or the surgical techniques employed throughout the study period.

### 2.2 Definitions

**Fig. 2.**
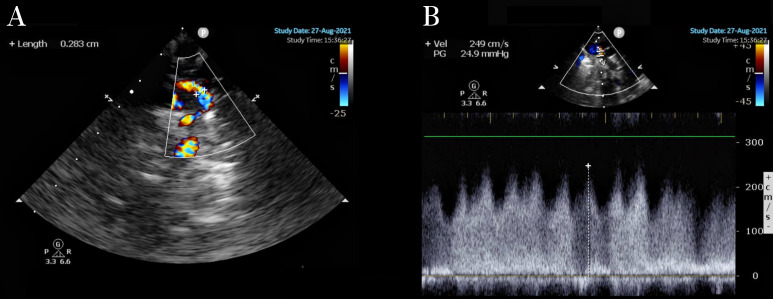
**Supracardiac TAPVC**. (A) Diameter of the obstructed vertical vein: 0.283 cm. (B) Vertical venous flow velocity 2.49 cm/s.

“Prolonged recovery” was defined by the ventilation duration. Prolonged recovery was defined as ventilatory support exceeding the 75th percentile. A TAPVC was characterized by a failure of the pulmonary venous confluence (PVC) to empty into the left atrium (LA) in combination with a persistent connection to the systemic venous circulation. A diagnosis of preoperative PVO was made by echocardiography, with a non-phasic flow velocity >1.8 m/s [8]. The measurement is normally recorded at the sites of obstruction. The blood flow in the pulmonary vein obstruction was turbulent, which was characterized by a continuous spectrum and the disappearance of the negative phase. The velocity was measured at the confluence, at the individual pulmonary veins, or in the vertical vein, as shown in Fig. 2. Heart failure was caused by pulmonary vein obstruction, leading to pulmonary congestion and pulmonary edema, which is characterized by hypoxemia (PaO_2_ <50 mmHg), metabolic acidosis, oliguria, circulatory failure, requiring mechanical ventilation with high positive end-expiratory pressure (PEEP) (>6 mmHg), and the use of catecholamine drugs. Surgery was performed when hypoxemia and heart failure could not be improved despite mechanical ventilation and pharmacotherapy.

A restrictive atrial septal defect (<3 mm) [7] was present when there was an obstruction at the interatrial septum. Severe PH was diagnosed when the velocity of tricuspid regurgitation was ≥3.5 m/s on the echocardiography [9]. TAPVC was categorized into four types according to the classification by Shaw and Chen [10]. Postoperative PVO was present when the peak blood flow velocity at the anastomosis of the vein and the left atrium was >1.8 m/s on the echocardiography [7, 8].

### 2.3 Preoperative Treatment

Children with severe PH and heart failure before surgery were treated with oxygen supplementation, positive inotropic drugs, diuretics, and correction of acidosis. Appropriate antibiotics were administered in cases with positive sputum cultures, as determined by next-generation sequencing (NGS). Invasive ventilator therapy was instituted for children with respiratory and circulatory failure characterized by respiratory distress, hypoxemia, hypercapnia, metabolic acidosis, and oliguria.

### 2.4 Operative Procedure

Endotracheal intubation and central venous and arterial catheters were inserted by an anesthesiologist before the operation. TAPVC repair was performed through a median sternotomy under standard aorto-bicaval cardiopulmonary bypass (CPB). The ductus arteriosus was dissected and ligated before initiating the CPB. Senior surgeons performed all operations. A sutureless technique was used when possible. Briefly, an incision was made in the venous confluence and then extended to the individual PV stenosis. *In situ* pericardial flaps were anastomosed distally to the incised veins to create a neo-LA. Detailed information on sutureless and conventional repair techniques has been previously published [7, 8, 9, 10, 11, 12, 13]. No infants underwent balloon atrial septostomy before surgical correction. Seven patients had left atrial catheters, and three patients had pulmonary artery catheters. All patients underwent either transthoracic or transesophageal ultrasound. Most newborns received a transthoracic ultrasound. Postoperative ultrasound in the operating room revealed significant anastomotic stenosis in four patients who required reoperation under CPB. Meanwhile, other concurrent cardiac anatomical issues, such as atrial septal defect (ASD), ventricular septal defect (VSD), patent ductus arteriosus (PDA), and coarctation of the aorta (COA), were also addressed during the pulmonary vein stenosis repair.

### 2.5 Postoperative Monitoring and ICU Management

All children were transferred to the CICU after surgery and were closely monitored for their heart rate, cardiac rhythm, blood pressure, central venous pressure, percutaneous oxygen saturation, body temperature, urine output, B-type natriuretic peptide (BNP), and blood–gas levels. Dopamine (5–7.5 µg⋅kg⋅min) and milrinone (0.25–0.75 µg⋅kg⋅min) were routinely used; meanwhile, epinephrine (0.05–0.1 µg⋅kg⋅min) or levosimendan (0.1–0.2 µg⋅kg⋅min for 24 hours) and norepinephrine (0.05–0.1 µg⋅kg⋅min) were used to improve cardiac output when necessary. Nitric oxide (NO) (5–20 ppm) inhalation, iloprost, bosentan, or phosphodiesterase-5 inhibitors were used alone or in combination for post-surgical management of reactive PH. For a mechanical ventilation time of <48 hours, midazolam (1–3 µg⋅kg⋅min) and dexmedetomidine (0.5–0.75 µg⋅kg⋅h) were administered; for a mechanical ventilation time of >48 hours, sedation and analgesia were provided with a combination of midazolam (2–4 µg⋅kg⋅min) and fentanyl (1–3 µg⋅kg⋅h, ≤1 month) or sufentanil (0.3–0.75 µg⋅kg⋅h, 2–6 month).

Echocardiography was performed at the bedside to assess the cardiac function and any residual defects. Subsequently, for children who exhibited an intraoperative pulmonary venous flow velocity of around 1.8 m/s, a postoperative ultrasound examination was performed on two consecutive days to obtain the average values of the pulmonary venous flow velocity, ejection fraction (EF), and left ventricular end diastolic dimension (LVDD).

The protocol for extubation after surgery required that children successfully pass a spontaneous breathing trial (SBT), which included stable hemodynamics, spontaneous breathing, urine output >1 mL/kg/hour, minimal chest tube output, and complete sternal closure. Other parameters included warm extremities, normal skin color, and a capillary refill of <3 seconds, a normal heart rate, palpable peripheral pulses, no lactic acidosis, an inotropic score <20, PEEP <6 cmH_2_O, FIO_2_ <60%, SpO_2_ target of >92% for all patients, a PaCO_2_ <45 mmHg, PaO_2_ >60 mmHg, and no acidosis. A total of 20 patients were re-intubated after a prior failed extubation.

When the hemodynamics of each patient were stable and ready for weaning, a bedside ultrasound examination of the diaphragm movement was performed. If one side of the diaphragm showed reduced movement, the patient was extubated, and non-invasive ventilation support was provided. For bilateral diaphragmatic weakness, neural-adjusted ventilator assist and potential monitoring were used. Ultimately, diaphragmatic plication was considered if the patient could not be weaned off non-invasive ventilation after extubation, had further elevation of the diaphragm or persistently low diaphragmatic potentials under neurally adjusted ventilatory assist (NAVA) monitoring, accompanied by atelectasis and weight loss caused by respiratory compromise.

A total of 18 children underwent diaphragmatic plication, and there were two cases of re-intubation due to arrhythmias after extubation.

### 2.6 Postoperative Follow-up

**Fig. 3.**
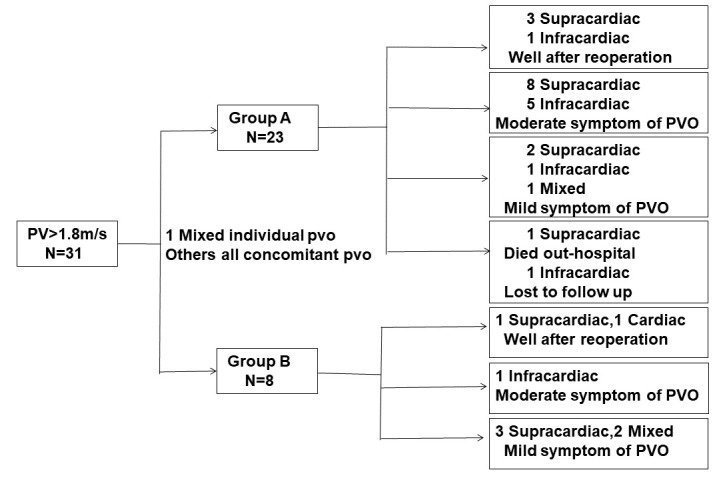
**Follow-up results in six months**. The children whose pulmonary vein flow velocities are >1.8 m/s after the operation. PV, pulmonary venous flow; PVO, pulmonary venous obstruction.

All surgical patients who were discharged from the hospital were required to return for outpatient follow-up visits at 1, 3, and 6 months after the initial operation. Some patients were required to visit local hospitals for routine examinations and follow-up if the patients were unable to return to our hospital. Any abnormal examination findings or changes in cardiac function resulted in a return visit for further evaluation. Both echocardiography and electrocardiography were routinely performed during the follow-up period. If the echocardiography Doppler finding indicated recurrent PVO, computed tomography (CT) was performed to evaluate the obstruction. In this study, 31 children had pulmonary vein flow velocities >1.8 m/s. The follow-up results at six months are detailed in Fig. 3. Moderate symptoms of PVO were defined as the need for oxygen and diuretic therapy. Mild symptoms were defined as requiring only diuretic treatment.

### 2.7 Data Collections

A preoperative echocardiogram was performed for all patients. Computed tomography angiography (CTA) was performed in selected children, as determined by the primary treating physicians, in 98% of patients (317 of 323) before surgery. A six-month follow-up was performed. Early postoperative death was defined as death during hospitalization or death within 1 month after discharge. Intermediate death was applied to refer to death more than 1 month after discharge.

The preoperative PV >1.8m/s, PH (tricuspid regurgitation (TR) ≥3.5m/s), age, weight, length, weight-for-age Z score (WAZ), LVDD Z value, sex, anatomic type, associated cardiac lesion, metabolic acidosis, emergency operation, respiratory tract infection within 2 weeks, and invasive mechanical ventilation were recorded. The CPB and aortic cross-clamp (ACC) times, delayed sternal closure, low cardiac output syndrome (LCO) within 48 h after the operation, velocity of pulmonary venous flow/ejection fraction (PV/EF) ratio, and velocity of pulmonary venous flow/left ventricular end diastolic dimension (PV/LVDD) ratio, diaphragmatic plication, sepsis, duration of ventilation, postoperative ICU and hospital stay, postoperative severe PH and PVO were all documented.

### 2.8 Statistical Analysis

Statistical analyses were performed using SPSS (version 22.0, IBM Corp, Armonk, NY, USA). Continuous data are presented as the median (range) or mean ± standard deviation (SD). Categorical data are presented as frequencies and percentages. The median and interquartile ranges of the postoperative mechanical ventilation period were calculated. Group A included children with a postoperative mechanical ventilation period longer than the 75th percentile of the postoperative mechanical ventilation period. Group B included the remaining children. A Student’s *t*-test or Wilcoxon–Mann–Whitney U test was used to compare the differences in the continuous data between the two groups. Categorical data were compared using the Pearson χ^2^ or Fisher’s exact test. A two-sided *p*-value < 0.05 was considered significant. Multivariate logistic regression analysis was used to identify factors associated with the prolonged recovery periods.

## 3. Results

### 3.1 Baseline Characteristics of Study Participants

A total of 369 children underwent TAPVC repair during the study period, of which 323 children were included in the analysis (Fig. 1). The median and interquartile ranges of the ventilation duration were 58 h and 120 h. During the operation, 311 (96.3%) children had moderate hypothermia, and 40 (12.4%) children required deep hypothermic circulatory arrest. The sutureless technique was used for 227 (70.3%) children. During the follow-up period, seven children required reoperation due to recurrent PVO. Among them, one child in group A refused reoperation and died two months after hospital discharge, while six children underwent reoperations, although recurrent PVO occurred during the 6-month follow-up period.

### 3.2 Bivariate Comparisons Between Two Groups

Table 1 summarizes the cohort characteristics. The median age at the time of surgery was 53.2 days (range: 0–180 days), and the median weight was 4.4 kg (range: 1.28–11.4 kg). Group A consisted of 66 children (38 males and 28 females) with a median age of 25.3 days, while group B included 257 children (156 males and 101 females) with a median age of 57.8 days. The preoperative, intraoperative, and postoperative characteristics were compared between the two groups (Tables 2,3,4).

**Table 1.** **Baseline characteristics of children included in the study (overall and stratified by hospital survivors)**.

Characteristics, N = 323		Measurements
Age, d		53.2 (0–180)
Weight, kg		4.4 (1.3–11.4)
Male, N (%)		194 (60.1)
Length, cm		55 (39–74)
Prematurity, N (%)		31 (9.6)
Mortality, N (%)		11 (3.3, 11/323)
Associated cardiac lesion, N (%)		
	Patent ductus arteriosus	241 (74.6)
	Atrial septal defect	323 (100)
	Ventricular septal defect	9 (2.8)
	Coarctation of aorta	3 (0.9)
	Ebstein	1 (0.3)
	Pulmonary artery stenosis/pulmonary artery branch stenosis	23 (7.1)
Anatomic type, N (%)		
	Supra-cardiac	153 (47.4)
	Cardiac	100 (31.0)
	Infra-cardiac	45 (13.9)
	Mixed	25 (7.7)

Note. The data are reported as the median (range) or n (%).

**Table 2.** **Comparison of preoperative characteristics between the two groups**.

Characteristics		Group A	Group B	*p*-value
		(N = 66)	(N = 257)	
Age, d		51.74 ± 19.8	55.47 ± 21.3	0.2
Newborns, N (%)		31 (47.0)	91 (35.4)	0.078
Weight, kg, mean ± SD		4.44 ± 1.51	4.7 ± 1.9	0.3
WAZ <−2, N (%)		20 (30.3)	12 (4.7)	<0.001
Male, N (%)		38 (57.6)	156 (60.7)	0.742
LVDD, Z value		−2.6 ± 1.23	−2.3 ± 0.91	0.15
Preoperative, PV >1.8 m/s				
	PV >1.8 m/s, N (%)	30 (45.5)	65 (25.3)	<0.001
	PV/EF (m/s/%)	3.14 ± 0.22	1.59 ± 0.20	<0.001
	PV/LVDD (m/s/cm)	1.44 ± 0.97	0.71 ± 0.07	<0.001
Supra-cardiac, N (%)		28 (42.4)	125 (48.6)	0.562
Cardiac, N (%)		17 (25.8)	83 (32.3)	0.339
Infra-cardiac, N (%)		19 (28.8)	26 (10.1)	0.03
Mixed, N (%)		3 (4.5)	22 (8.6)	0.288
PH (TR ≥3.5 m/s), N (%)		46 (69.7)	135 (52.5)	0.015
Preoperative infection, N (%)		16 (24.2)	36 (14.0)	0.074
Preoperative intubation, N (%)		19 (28.8)	45 (17.5)	0.064
Metabolic acidosis, N (%)		13 (19.7)	34 (13.2)	0.282
Emergency operation, N (%)		36 (54.5)	72 (28.0)	<0.001

Note. WAZ, weight-for-age Z score; LVDD, left ventricular end diastolic dimension; PV, pulmonary venous flow; EF, ejection fraction; PH, pulmonary hypertension; TR, tricuspid regurgitation; SD, standard deviation. The data are reported as the median (range), mean ± SD, or n (%).

**Table 3.** **Comparison of intraoperative characteristics between the two groups**.

Characteristics		Group A	Group B	*p*-value
		(N = 66)	(N = 257)	
Associated cardiac lesion				
	PAS, N (%)	5 (7.6)	18 (7.0)	0.745
	VSD, N (%)	1 (1.5)	2 (0.8)	0.185
	COA, N (%)	2 (3.0)	7 (2.7)	0.834
CPB time, min, mean ± SD		130 ± 70.39	84.5 ± 27.6	<0.001
ACC time, min, mean ± SD		65.0 ± 31.9	49.6 ± 22.0	0.008
Use of sutureless repair, N (%)		47 (71.2)	181 (70.4)	0.928
Delayed sternal closure, N (%)		22 (33.3)	54 (21.0)	0.054
Operations per year by a senior surgeon, cases, mean ± SD		232 ± 78	235 ± 82	0.521

Note. PAS, pulmonary artery stenosis; VSD, ventricular septal defect; COA, coarctation of the aorta; CPB, cardiopulmonary bypass; ACC, aortic cross-clamping. The data are reported as actual values, means ± SD, or n (%).

**Table 4.** **Comparison of postoperative characteristics between the two groups**.

Characteristics		Group A	Group B	*p*-value
		(N = 66)	(N = 257)	
LCOS, N (%)		26 (39.4)	62 (24.1)	0.02
	P(v–a)CO_2_, mmHg	8.2 (5.5–14.8)	5.3 (3.0–8.5)	0.03
	S(a–v)O_2_, %	21.3 (12.7–34.9)	18.2 (13.2–25.8)	0.29
	ScvO_2_, %	60.5 (44.2–67.6)	71.6 (64.1–81.3)	0.04
	Lac, mmol/L	4.3 (2.0–10.2)	2.4 (1.6–4.3)	0.02
	BNP (pg/mL)	5236 ± 871.8	7807 ± 2484.1	0.159
	IS	17.7 ± 1.8	12.3 ± 1.2	0.03
PH (TR ≥3.5 m/s), N (%)		18 (27.3)	50 (19.5)	0.08
Infection, N (%)		15 (22.7)	35 (13.6)	0.112
Diaphragmatic plication, N (%)		16 (24.2)	2 (0.78)	<0.001
Duration of ventilation, h		182 (129–235)	52 (27–78)	<0.001
Po-op ICU stay, d		12 (9–18)	5.5 (3.5–10.5)	<0.001
PV >1.8 m/s				
	PV >1.8 m/s, n (%)	9 (13.6)	22 (8.56)	0.353
	PV/EF (m/s/%)	1.93 ± 0.18	1.52 ± 0.09	0.066
	PV/LVDD (m/s/cm)	0.69 ± 0.07	0.51 ± 0.04	0.018
NIV within 48 h of extubation, N (%)		45 (69.2)	125 (48.6)	0.003
NIV time, h, mean ± SD		62.7 ± 23.9	42.2 ± 15.0	<0.001
Po-op hospital stay, d, mean ± SD		16.5 ± 4.5	9.5 ± 2.4	<0.001

Note. LCOS, low cardiac output syndrome; P(v–a)CO_2_, partial pressure of carbon dioxide between the artery and vein; S(a–v)O_2_, arterial–venous oxygen saturation difference; ScvO_2_, oxygen saturation of the central vein; Lac, lactate; BNP, brain natriuretic factor or peptide; PH, pulmonary hypertension; Po-op, post-operation; PV, pulmonary venous flow; LVDD, left ventricular end diastolic dimension; EF, ejection fraction; IS, positive inotropic drug score; NIV, non-invasive ventilation. The data are reported as the median (range), mean ± SD, or n (%).

### 3.3 Preoperative Analysis

In the preoperative analysis, the children in group A had significantly more intracardiac anatomic types, a higher WAZ <–2 score, and more severe PH and emergency operation compared with the children in group B. Moreover, the children in group A had statistically significantly higher PV values >1.8 m/s, PV/EF, and PV/LVDD than the children in group B (Table 2).

### 3.4 Intraoperative Analysis

In the intraoperative analysis, statistically significant differences were observed in CPB time and ACC time (Table 3). Conversely, there were no differences between the two groups in terms of other congenital heart defects, surgeon experience, surgical method, or the proportion of delayed chest closure postoperatively in the intraoperative analysis. The children in group A had longer CPB and ACC times than the children in group B.

### 3.5 Postoperative Analysis

**Fig. 4.**
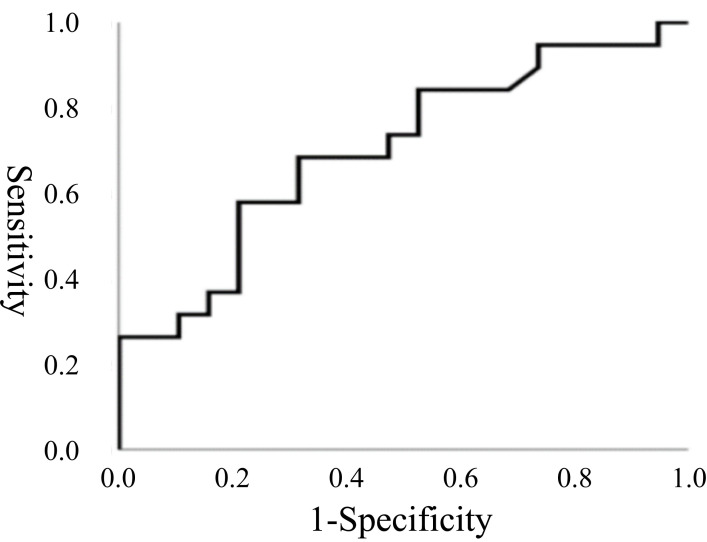
**Receiver operating characteristic (ROC) curve for the PV/LVDD**. ROC curve for the PV/LVDD on the first day after the operation. The area under the full ROC curve is 0.702, with a specificity of 0.789 and an estimated sensitivity of 0.579. The PV/LVDD cutoff value was 0.624.

Group A had a higher incidence of low cardiac output syndrome (LCOS) and required more diaphragmatic plications than Group B. LCOS was characterized by an elevated partial pressure of carbon dioxide (P(v–a)CO_2_) between the artery and vein, decreased central venous oxygen saturation (ScvO_2_), increased lactate levels, and a higher positive inotropic drug score (IS). Children in group A required longer durations of non-invasive ventilation (NIV) support than those in group B. The PV/LVDD ratio was significantly higher in group A, with a cutoff value of 0.624 identified for prolonged postoperative recovery (Fig. 4). Of the 11 children who died postoperatively, 10 died in the hospital, and one died two months after discharge. Four of these children had a PV of >1.8 m/s, and nine had a PV/LVDD ratio of >0.624. All infections listed in Table 4 were new-onset postoperative infections, detected through postoperative sputum culture, sputum NGS examination, or blood culture tests.

### 3.6 Multivariate Logistic Regression Analysis

The multivariate analysis revealed that a WAZ <–2, preoperative PVO, emergency surgery, prolonged CPB and ACC times, postoperative diaphragmatic injury, and PV/LVDD were independent risk factors for a protracted recovery period (Table 5); however, intracardiac and LCOS were not statistically significant. A PV of >1.8 m/s, PV/EF, PV/LVDD, and preoperative PH before surgery were statistically significant between the two groups (Table 2); however, only PV/LVDD remained statistically significant after surgery.

**Table 5.** **Multivariate logistic regression analysis for prolonged recovery**.

Variables	B	Standard error	Wald Chi-Square	*p*-value	Exp (B)	95% confidence interval
WAZ <−2	1.847	0.756	5.996	0.022	6.358	1.546–27.845
Preoperative PVO	1.79	0.880	4.133	0.042	5.987	1.066–33.617
Emergency operation	1.776	0.876	4.114	0.043	5.906	1.062–32.853
CPB/ACC time	2.151	0.877	6.014	0.014	8.591	1.540–47.927
Diaphragmatic plication	1.676	0.836	4.020	0.045	5.346	1.038–27.519
PV/LVDD	2.112	0.845	6.249	0.012	8.265	1.578–43.289

Note. WAZ, weight-for-age Z score; PVO, pulmonary venous obstruction; LVDD, left ventricular end diastolic dimension; PV, pulmonary venous flow; CPB, cardiopulmonary bypass; ACC, aortic cross-clamping. The data are reported as the median (range), mean ± SD, or n (%).

## 4. Discussion

Before the 1970s, the postoperative mortality rate for infants with TAPVC was as high as 50%. However, advances in extracorporeal circulation, surgical techniques, anesthesia, and perioperative care have significantly reduced mortality rates. Seale *et al*. [14] reported an in-hospital mortality rate of 14.3% in a retrospective analysis of 406 children with TAPVC across 19 centers of the British Congenital Heart Association from 1998 to 2004. Similarly, Lemaire *et al*. [15] found that the total mortality rate in 180 children with TAPVC decreased from 42.1% to 7.4% between 1973 and 2014. A study from Shanghai Children’s Medical Center and the Guangdong Institute of Cardiovascular Diseases reported an early postoperative mortality rate of 5% and a mid-postoperative mortality rate of 2% in 768 children with TAPVC who underwent surgical correction between 2005 and 2014 [7]. Comparatively, there was an overall mortality rate of 3.3% among 323 children with TAPVC in the present study, with 10 early deaths and one mid-term death. Notably, all 11 children who died had preoperative PVO, and half were in critical condition before surgery, necessitating emergency intervention. In this study, emergency operation increased the likelihood of prolonged ventilation by 5.9-fold. Group A had a higher incidence of preoperative PVO, emergency surgeries, and one mid-term death, underscoring the importance of thorough preoperative evaluation and prompt surgical intervention.

Our previous research has shown that prolonged CPB time and ventilation duration in neonatal patients are associated with delayed recovery [7]. Furthermore, this study aligns with prior data, which show that an extended extracorporeal circulation time increases the likelihood of prolonged ventilation by 8.6-fold. The present study showed that the mortality rate of infants younger than 6 months with TAPVC treated in our hospital in the past four years was further reduced to 3.3%. In the multivariate analysis, a WAZ <–2 was associated with delayed recovery; however, age and weight exhibited no association. A WAZ <–2 had a 6.4-fold higher likelihood of prolonged recovery. Our previous research has demonstrated that hemodynamic factors are risk factors for malnutrition after surgery [16]. This emphasizes the role of nutritional status in postoperative outcomes, as a WAZ <–2 and diaphragmatic injury were significant predictors of delayed recovery. Despite these challenges, the follow-up findings showed that these children generally recovered well, demonstrating improved weight and a stable respiratory function.

A 5.3-fold higher likelihood of a prolonged recovery was observed in patients who underwent diaphragm plication. Nonetheless, 18 infants underwent diaphragm plication, all of whom had positive outcomes, highlighting the effectiveness of minimally invasive techniques in managing diaphragmatic injury, particularly in very young patients who struggle to be weaned off ventilation [17]. Preventing diaphragmatic injury and ensuring adequate circulatory and respiratory support postoperatively are crucial. To manage PH, therapies such as NO inhalation, iloprost, bosentan, or phosphodiesterase-5 inhibitors were utilized, either alone or in combination. Although preoperative severe PH differed between the two groups, no significant differences in postoperative PH were observed. This suggests that preoperative PH is primarily related to PVO; meanwhile, early postoperative PH may result from factors, such as reactive pulmonary vasoconstriction, which are induced by CPB or anatomical obstructions in pulmonary venous return. Non-invasive ventilation support after weaning was more frequently required in group A, and improved cardiopulmonary function and facilitated successful weaning.

Although the postoperative survival rate of children with TAPVC has significantly improved recently, the early postoperative mortality of children younger than six months with TAPVC in most cardiac centers has not been significantly improved. While performing surgery on children with TAPVC is challenging, it is recommended to conduct the procedure as soon as possible [18]. A total of 108 patients underwent emergency surgery in this study, with most achieving a good recovery in the CICU. Meanwhile, a combined 76 cases of delayed sternal closure were noted in the two groups. Delayed sternal closure was performed to accommodate the normalization of right ventricular work and stabilization of PA pressure and relieve myocardial edema and LCOS.

Traditional indicators, such as prolonged CPB time and longer ACC time, were confirmed as risk factors for delayed recovery [19, 20]. Additionally, a higher PV/LVDD ratio emerged as a novel indicator. While a PV >1.8 m/s was used to define PVO preoperatively, its significance diminished postoperatively, unlike the PV/LVDD ratio, which remained a robust predictor with a cutoff value of 0.624. The PV/LVDD ratio offers a comprehensive assessment of pulmonary venous flow and left ventricular end-diastolic volume, reflecting early pulmonary vein conditions and predicting delayed recovery, worse prognosis, and even mortality. Among the 11 patients who died, 4 had a PV >1.8 m/s, and 9 had a PV/LVDD ratio >0.624, underscoring the predictive value of this ratio. Preoperative and postoperative pulmonary vein obstruction often leads to right ventricular enlargement, with a relatively smaller left atrium and left ventricle. However, the status of the left atrium and left ventricle improves after resolving pulmonary vein obstruction. Compared to single PV flow velocity, the PV/LVDD ratio can provide a more comprehensive and long-term assessment of the pulmonary vein obstruction status in each child. In this study, PV/LVDD had a 9.8-fold higher likelihood of prolonged recovery. Additionally, LVDD is easily obtainable and repeatable on ultrasound examinations. Thus, this index can accurately assess the state of pulmonary vein obstruction and can serve as another reliable indicator for pulmonary vein obstruction.

A perioperative recovery study in a large sample of infants in the ICU is significant for improving the perioperative management of these patients. The study emphasizes the importance of nutritional support and maintaining normal perioperative respiratory and cardiac hemodynamic function for these infants, thereby avoiding emergency surgeries, and the benefits of sequential support with non-invasive ventilators after invasive ventilator withdrawal for high-risk infants with delayed recovery. Importantly, preventing injury to the diaphragm remains crucial; thus, early detection and assessment of diaphragmatic injury, as well as determination of whether diaphragmatic plication is necessary, are vital. Indeed, for patients with high PV flow rates, it is advisable to actively manage volume overload, adequately treat pulmonary infections, and follow up on the improvement of PV/LVDD, which reflects the left and right ventricular diastolic function.

Our previous studies by Doctor Shi *et al*. [7] and Doctor Shentu *et al*. [21] reviewed surgical techniques and the importance of perioperative monitoring. This study primarily focused on intensive care recovery strategies for TAPVC patients under the age of six months. Other studies also reported postoperative recovery metrics and associated risk factors in TAPVC patients [22, 23, 24, 25]. A study by the Children’s Hospital, Zhejiang University School of Medicine, showed that postoperative mechanical ventilation was an independent risk factor for prolonged ICU stay in 85 children with TAPVC [24]. Meanwhile, a study by the Children’s Hospital of Chongqing Medical University on 54 patients with TAPVC found that emergency surgery may be associated with a prolonged ICU stay [25]. Our research involves a larger sample size and includes some new perspectives.

However, this study has several limitations that should be acknowledged. First, the retrospective design possesses an inherent risk of selection bias, as well as information bias due to the reliance on existing medical records. Second, the study was conducted at a single center, which may limit the generalizability of the findings to other populations or healthcare settings. Third, the study focused exclusively on short-term postoperative outcomes, with the follow-up limited to six months. This short follow-up period may not capture long-term complications or outcomes, such as late-onset pulmonary venous obstruction or reoperations, which are important in the context of TAPVC management. Additionally, this study did not include a control group of patients who underwent other forms of treatment, which could have provided comparative insights. Lastly, the use of the PV/LVDD ratio as a predictor of prolonged recovery is novel; however, this ratio requires further validation in larger, multicenter studies to establish its robustness and clinical utility.

## 5. Conclusions

In summary, a WAZ score <–2, preoperative pulmonary venous obstruction, emergency surgery, prolonged CPB and ACC time, diaphragmatic injury, and PV/LVDD ratio >0.624 were associated with a higher risk of prolonged recovery in infants with TAPVC. The PV/LVDD ratio, with a cutoff value of 0.624, has emerged as a promising marker for identifying those at risk of prolonged recovery. For high-risk patients, perioperative nutritional optimization is also important since a lower WAZ indicates a delayed recovery. Precise surgical technique, controlled CPB time, and prevention of diaphragmatic injury are very important. Early surgery is required when the diagnosis is confirmed to avoid emergency surgery and postoperative delayed recovery.

## Availability of Data and Materials

The datasets used and analyzed in the current study are available from the corresponding author upon reasonable request.
